# Feed efficiency and fecal microbiome of nursery pigs from parents with divergent breeding value for feed conversion ratio

**DOI:** 10.1093/tas/txaf026

**Published:** 2025-02-20

**Authors:** Yujia Wu, Paula Azevedo, Shunshun Jin, Haoxiang Xu, Huaigang Lei, Lisanne Verschuren, Argenis Rodas-Gonzalez, Martin Nyachoti, Chengbo Yang

**Affiliations:** Department of Animal Science, University of Manitoba, Winnipeg, Manitoba, R3T 2N2, Canada; Department of Animal Science, University of Manitoba, Winnipeg, Manitoba, R3T 2N2, Canada; Department of Animal Science, University of Manitoba, Winnipeg, Manitoba, R3T 2N2, Canada; Department of Animal Science, University of Manitoba, Winnipeg, Manitoba, R3T 2N2, Canada; Department of Animal Science, University of Manitoba, Winnipeg, Manitoba, R3T 2N2, Canada; Topigs Norsvin Canada Inc., Oak Bluff, Manitoba, R4G 0C4, Canada; Topigs Norsvin Central, Meerendonkweg 25, Den Bosch, the Netherlands; Department of Animal Science, University of Manitoba, Winnipeg, Manitoba, R3T 2N2, Canada; Department of Animal Science, University of Manitoba, Winnipeg, Manitoba, R3T 2N2, Canada; Department of Animal Science, University of Manitoba, Winnipeg, Manitoba, R3T 2N2, Canada

**Keywords:** breeding value, digestibility, gut microbiota, performance traits, predicted functionality

## Abstract

Improving feed efficiency (FE) is essential for the swine industry’s economic and environmental sustainability. Genetic selection, particularly through estimating breeding values for feed conversion ratio (EBV_FCR), is a common strategy to enhance FE. However, the biological mechanisms underlying phenotypic variations in FE between pigs with different EBV_FCR values are not fully understood. This study investigates these mechanisms by examining growth performance, nutrient and energy digestibility, and fecal microbiota composition and functionality of pigs at the nursery stage. The study involved 128 pigs, weaned at 21 d (±2 d) and with an initial body weight of 6.87 kg (±0.34 kg). These pigs, selected from dam and sire lines with divergent EBV_FCR values, were randomly assigned to 32 pens with four pigs each. Pigs were fed a corn and soybean meal-based diet, divided into two feeding phases of 2 wk each, under similar rearing conditions. Results indicated no significant differences in average daily feed intake (ADFI), average daily body weight gain (ADG), or feed efficiency (FE, gain:feed) between pigs from different EBV_FCR lines (*P* > 0.05). Similarly, nutrient digestibility showed no significant variation (*P* > 0.05). While the overall fecal microbiota taxonomic composition was similar between the groups, there was a trend toward higher beta diversity in the microbiota of pigs from parents with lower EBV_FCR (high efficiency pigs, H pigs) (*P* < 0.083). Carbohydrate and amino acid metabolism were predominant in all pigs, regardless of genetic background, with similar predicted microbiota functionality across groups. The study concluded that genetic differences based on parents divergent EBV_FCR did not affect growth performance, nutrient utilization, or microbiota characteristics at the nursery stage. This suggests that while EBV_FCR based genetic selection does not impact early-stage performance or microbiome responses, its effects may differ in older pigs, warranting further research.

## INTRODUCTION

Feed costs in swine production can account for 60% to 70% of total expenses, making feed efficiency (FE) a critical factor for improving economic and environmental sustainability in the industry (McCormack et al., 2017). One approach to enhancing FE is selecting pigs with low estimated breeding value (EBV) for feed conversion ratio (FCR), where a negative EBV for both dam and sire lines is desirable as it indicates lower FCR, and therefore higher FE in offspring. Pigs with low EBV for FCR typically show better feed efficiency by achieving the same body weight gain with reduced feed intake (Verschuren et al., 2021). In grower pigs, higher fecal digestibility was associated with improved FE and lower residual feed intake (RFI) (Verschuren et al., 2021). However, it remains unclear whether similar FE differences occur in post-weaning nursery pigs from parents with divergent EBV_FCR, especially in terms of early growth performance, nutrient utilization, and digestibility.

The gut microbiota is an important factor influencing FE, as it plays a significant role in energy harvest, nutrient digestion, and intestinal health (McCormack et al., 2017). Studies have shown that gut microbiome composition differs between pigs with varying FE. Specific bacterial families and genera, such as *Christensenellaceae*, *Actinobacillus*, *Treponema*, and *Methanobrevibacter*, are associated with high FE pigs (Gardiner et al., 2020). Other genera, such as, *Streptococcus* and *Desulfovibrio*, have also been shown to be correlated with lower RFI and higher FE (Aliakabari et al., 2021). However, little is known about the gut microbiome of post-weaning nursery pigs from parents with divergent EBV_FCR, especially since weaning induces significant shifts in gut microbiota due to stress associated with weaning and due to the introduction of solid feed among other changes (Wang et al., 2019).

Next-generation sequencing technologies, particularly 16S rRNA gene sequencing, offer a powerful tool for characterizing microbial communities and understanding their relationship with FE (Caporaso et al., 2010; Chong et al., 2020). This study hypothesizes that differences in FE among nursery pigs from parents with high or low EBV_FCR can be explained by variations in nutrient digestibility and gut microbiota composition and function. The objectives were to: 1) compare growth performance, nutrient, and energy digestibility of nursery pigs from parents with divergent EBV_FCR; and 2) characterize the composition and predicted functionality of their fecal microbiome.

## MATERIALS AND METHODS

The experiment and all measurements were approved by the Animal Care Committee of the University of Manitoba (AC#F21-002), and pigs were handled according to the guidelines described by the Canadian Council on Animal Care (CCAC, 2009).

### Animals and Experimental Design

The study included a total of 128 piglets (initial body weight, IBW = 6.87 ± 0.34 kg (±SD)) and weaning age (21 d ± 2 d) obtained from the Glenlea Research Station. Castrated males and female piglets were three-way crossbred offspring (Synthetic boar × (Landrace × Large White)) from Topigs Norsvin (Oak Bluff, Manitoba, Canada). The EBVs of individual pigs were estimated using the Single Step Genomic Best Linear Unbiased Prediction (ssGBLUP) method in Mixed Best Linear Unbiased Prediction (MIXBLUP) (Vandenplas et al., 2023). A 25K single-nucleotide polymorphism chip was used to create the genomic relationship matrix and the EBVs for FCR were estimated by Topigs Norsvin Research Center (Beuningen, The Netherlands), using data collected over the past 10 yr. The piglets were housed at the TK Cheng Centre at the University of Manitoba to a total of 32 pens (4 animals per pen and per 2.88 m^2^) with one feeder in each pen and unlimited access to water. Piglets were randomly assigned to each pen. Piglets were fed ad libitum. From weaning at day 21(±2) (pig’s age), piglets were fed a pre-starter diet (NE of 2.58 Mcal/kg: 1.35% SID lysine), and from day 42 to day 56, piglets were fed a starter diet (NE of 2.44 Mcal/kg: 1.20% SID lysine). The high efficiency (H) piglets were obtained by inseminating low EBV_FCR sows (−0.12 ± 0.011) with semen from low EBV_FCR boars ((−0.29 ± 0.041), and the low efficiency (L) piglets were obtained by inseminating high EBV_FCR sows (0.0 ± 0.027) with semen from high EBV_FCR boars (0.033 ± 0.045). All EBV_FCRs were provided by Topigs Norsvin based on their routine genetic evaluation.

### Experimental Diets

The experimental diet was a corn-soybean meal-based diet in a 2-phase feeding program (Table 1) for 4 wk. Ingredients were selected based on relevance to the swine industry in Manitoba (Canada) and followed the breeding line requirements (Topigs Norsvin) and at the same time meet or exceed the NRC (2012) recommendations for nursery pigs.

**Table 1.** Composition of experimental diets, as-fed basis (g/kg)

Ingredient	Pre-starter (7–11 kg)	Starter (11–25 kg)
Corn	257.42	418.38
Wheat	200	225
Soybean meal	152	201
Canola meal	0	25
Fish meal	55	20
Dried whey powder	110	35
Vegetable oil	29.5	9
Oat groats	100	0
Barley	0	25
Hamlet HP300^1^	40	15
NUPRO^2^	30	0
Betaine	2	0
Zinc oxide	3.47	0
Limestone	4	7
Dicalcium phosphate	0	3.0
Salt	3.75	4.25
Copper sulfate	0.5	0.5
Vitamin premix^3^	1.5	1.5
Mineral premix^4^	1	1
Choline chloride (70%)	0.8	0.5
_ L_-Lysine-HCl	4	4.25
_ DL_-Methionine	2.23	1.8
_ L_-Threonine	1.52	1.66
_ L_-Tryptophan	0.76	0.54
_ L_-Valine	0.45	0.52
Phytase^5^	0.1	0.1
**Calculated Composition**		
Dry Matter, %	89.53	87.68
NE^6^ Swine Calc, Mcal/kg	2.58	2.44
Crude Protein, %	21.88	19.75
Crude Fat, %	6.13	3.77
Crude Fiber, %	1.86	2.4
ADF^7^, %	2.01	3.22
NDF^8^, %	6.39	9.47
SID^9^ Lysine, %	1.35	1.2
SID^9^ Methionine, %	0.53	0.45
SID^9^ Tryptophan, %	0.28	0.24
SID^9^ Threonine, %	0.88	0.78
Available Calcium, %	0.89	0.76
Available Phosphorus, %	0.81	0.67
SID Lys/NE Ratio	5.23	4.91
** Analyzed Composition**		
Dry matter, %	89.3	88.6
Crude protein, %	22.3	20.8
Crude fat, %	5.47	3.33
Crude fiber, %	1.64	2.15
ADF, %	5.14	5.65
NDF, %	10.43	11.91
Ash, %	5.42	5.05
Ca, %	0.59	0.57
P, %	0.65	0.59

Hamlet HP300^1^: from Hamlet Protein; NUPRO^2^: from Alltech.

Vitamin premix^3^ and mineral premix^4^, from DSM, provided per kg of diet: Vitamin A, 13,5000 IU; Vitamin D_3_, 750 IU; 25-OH-D3, 1000 IU; Vitamin E, 135 IU; Vitamin K, 4.00 mg; Vitamin B_1_, 4.00 mg; Vitamin B_2_, 11.02 mg; Vitamin B_3_, 50.00 mg; Vitamin B_5_, 45.00 mg; Vitamin B_6_, 4.5 mg; Vitamin B_12_, 55.00 mcg; Vitamin B_9_, 1.67 mg; Vitamin C, 100.00 mg; Vitamin B_7_, 300.00 mg; Fe, 140.00 ppm; Cu, 25.00 ppm; I, 1.00 ppm; Se,0.30 ppm; Mn 75.00 ppm; Zn, 130.00 ppm.

Phytase^5^: from AB Vista. NE^6^: Net energy; ADF^7^: Acid detergent fiber; NDF^8^: Neutral detergent fiber; %SID^9^: standardized ileal digestible.

### Growth Performance Measurements and Sampling

At the beginning of the trial and at the end of the pre-starter and starter feeding phases, piglets were individually weighed, and feed intake per pen (experimental unit) for the 2 wk of each phase was measured, and FE (gain:feed) for each feeding phase and the overall feeding trial was calculated. At the end of the feeding phase 1, eight piglets (BW = 16.01 ± 0.57 kg) from each group were randomly selected and moved into individual metabolic crates (1.8 × 0.6 m) to determine the total tract digestibility of crude protein, dry matter, gross energy, crude fat, and crude fiber. Room temperature was controlled to 25 ± 2 °C. The screen collected feces under the crate. The pigs stayed in crates for 10 d of adaptation and 6 d of collection. Pigs had unlimited access to water and were fed once daily at 6 a.m. Feed amounts offered were set to 550 kcal ME/kg BW^0.60^ to be close to ad libitum (Noblet et al., 1994), and pigs were weighed every 5 d to adjust the feed amount.

After the adaptation period, a marker (ferric oxide) was mixed with the feed on day 1 and day 6 of collection days. Feces samples were collected according to the SOPs from the Computarized Laboratory Animal Monitoring System (CLAMS, University of Manitoba) once the marker first appeared until the marker added on day 6 disappeared to determine digestible nutrient and energy contents of the diet. The fecal samples were weighed and stored in a −20 °C freezer.

At the end of the feeding trial, fresh fecal samples from pigs in each pen were collected by grab sampling feces as soon as pigs defecated and stored at −80 °C for microbiome analysis. Also, 8 pigs from each group were randomly selected and anaesthetized by Ketamine: xylazine (20:2 mg/kg BW) and killed by a captive bolt gun. The spleen, heart, lung, kidneys, liver, and stomach were taken for organ weight, and the length of the large and small intestine were measured.

### Chemical Analysis

Diets, and fecal samples were analyzed by a commercial laboratory, Central Testing Lab (Winnipeg, MB, Canada). Before chemical analysis, the fecal samples were dried in a forced-air drying oven at 60 °C for 7 d, pooled for each pig, and finely ground. Diets and fecal samples were analyzed for dry matter (DM), gross energy (GE), crude protein (CP), crude fat, starch, neutral detergent fiber (NDF), acid detergent fiber (ADF), acid insoluble ash (AIA), and calcium (Ca) and phosphorus (P). The moisture (AOAC 930.01), crude protein (AOAC 990.03), Ash (AOAC 942.05), crude fat (AOAC AM5-04), Ca and P (AOAC 985.01), crude fiber (AOAC Ba6a-05) and AIA (AOAC 942.05) were determined according to the methods of the Association of Official Analytical Chemists International (AOAC International, 2006). The starch content was measured using the amyloglucosidase/alpha-amylase method (AOAC 996.11) using a starch megazyme starch kit. The ADF and NDF contents were analyzed according to the method by Goering (1970) using an ANKOM 200 Fiber Analyzer (A200, ANKOM Technology, Macedon, NY) with an alpha-amylase (product A3306, Sigma-Aldrich, St. Louis, MO). The GE was determined using a bomb calorimeter (model 6400, Parr Instruments Co, Moline, IL) calibrated using benzoic acid as a standard.

### Calculations

Apparent total tract digestibility (ATTD) of DM, GE, CP and energy were calculated using the following formula:


$$\begin{array}{l} ATTD\;\left(\%\right)=\frac{\left[C\;input-C\;output\right]}{C\;input}\times 100 \end{array}$$

where C input and C output are the amount of nutrient or energy ingested and voided via the feces, respectively (Kong and Adeola., 2014).

The relative organ weight was calculated as:


$$\begin{array}{l} Relative\;organ\;weight\;\left(\%\right)=100\times \left[organ\;weight\left(kg\right)/total\;pig\;weight\left(kg\right)\right] \end{array}$$

### DNA Extraction and 16S rRNA Sequencing

The fecal DNA was extracted using the QIAamp® Fast DNA Stool Mini Kits (Qiagen Ltd., Germany) according to the manufacturer’s instructions, and bead-beating was included to lyse the microbial cells. The quantity and quality of extracted DNA were measured using a NanoDrop2000 spectrophotometer (Thermo Fisher Scientific, Waltham, MA, USA) and agarose gel electrophoresis, respectively. DNA samples were sent to Argonne National Laboratory for sequencing. The V4 region of the 16S rRNA gene was amplified with universal primers 515F (GTGYCAGCMGCCGCGGTAA) (Parada et al., 2016) and 806R (GGACTACNVGGGTWTCTAAT) (Apprill et al., 2015). Illumina MiSeq 250-bp paired-end reads were used to obtain the full-length reads of the V4 region. Each 25 µL PCR (polymerase chain reaction) reaction contained 9.5 µL Certified DNA-Free, 12.5 µL of QuantaBio’s AccuStart II PCR ToughMix (2x concentration, 1x final), 1 µL Golay barcode tagged forward primer (5 µM concentration, 200 pM final), 1 µL reverse primer (5 µM concentration, 200 pM final), and 1 µL of template DNA. The conditions for PCR were as follows: 94 °C for 3 min to denature the DNA, with 35 cycles at 94 °C for 45 s, 50 °C for 60 s, and 72 °C for 90 s; with a final extension of 10 min at 72 °C to ensure complete amplification. Amplicons were then quantified using PicoGreen (Invitrogen) and a plate reader (Infinite® 200 PRO, Tecan). Once quantified, volumes of each of the products were pooled into a single tube so that each amplicon was represented in equimolar amounts. This pool was then cleaned up using AMPure XP Beads (Beckman Coulter), and then quantified using a fluorometer (Qubit, Invitrogen). After quantification, the molarity of the pool was determined and diluted down to 2 nM, denatured, and then diluted to a final concentration of 6.75 pM with a 10% PhiX spike for sequencing on the Illumina MiSeq. Amplicons were sequenced on a 251 bp × 12 bp × 251 bp MiSeq run using customized sequencing primers and procedures (Caporaso et al., 2012). Reads were joined using EA-Util’s fastq-join script with default parameters, then screened to exclude sequences that contained one or more base calls with a Phred quality score of less than 20. A Phred quality score of 20 or higher indicated an accuracy of 99%.

### Microbial Data Analysis

Raw sequences were analyzed using the latest version of the QIIME2 platform (version 2021.8) as previously described by Wang et al. (2019). Initial reads were quality filtered, denoised, assembled, and chimeric sequences were removed using DADA2 (Callahan et al. 2016), which generates unique amplicon sequence variants (ASVs). Samples were rarefied to the same number of reads of 30,000 sequences per sample, the smallest number of sequences across all collected samples, before downstream analysis. We used the SILVA (version 138) reference database classifier to classify bacterial features with a threshold of 99% sequence similarity. Alpha and beta diversities were calculated in QIIME2. To examine the effects of pigs’ genetics on fecal microbiota, we performed a permutational multivariate analysis of variance (PERMANOVA, with 999 Monte Carlo permutations) based on Bray- Curtis, Unweighted Unifrac distances, and Weighted Unifrac distances matrices with the web-based tool MicrobiomeAnalyst2 (Chong et al., 2020). Differentially abundant features (genus level) between pig groups were identified using the DESeq2 method by Love et al. (2014) using relative log expression (RLE) normalization of the data (Anders and Huber, 2010). Data visualization was performed using the ggplot2 package in R (version 4.22) and MicrobiomeAnalyst 2 (Chong et al., 2020). The predicted metagenomes and function of the gut microbiota were inferred using an R package Tax4FUN (Aßhauer et al., 2015) available through MicrobiomeAnalyst2. The predicted genes and their function were aligned to the Kyoto Encyclopedia of Genes and Genomes (KEGG) database, and the differences between pig groups were compared through the software STAMP https://beikolab.cs.dal.ca/software/STAMP (Parks and Beiko, 2010). Two-side Welch’s t-test and Benjamini-Hochberg false discovery rate (FDR) correction were used in two-group analysis.

### Statistical Analysis

Growth performance data were analyzed using PROC MIXED in SAS 9.4 (SAS Inst. Inc., Cary, NC, United States). The model included fixed effect of pig genetics and pen as a random effect. The pen was the experimental unit. Pigs were randomly assigned to pens regarding their weaning age, initial body weight and sex. The covariate effects of weaning age, initial body weight (IBW), and sex (ratio of castrated males/females) were tested and their effects were not significant and therefore removed from the model. A *P* value below 0.05 was considered statistically significant, while a *P* value between 0.05 and 0.1 indicates a trend for differences. A pen was the experimental unit for body weight gain (ADG), feed intake (ADFI), and FE data. For organ weight and length data and digestibility data, the mixed procedure of SAS was also used, but, in this case, the individual pig was the experimental unit. Unpaired T-test analysis was performed to compare performance and organ measurements as well as digestibility between the 2 pig groups. For the microbiome data, all parametric data were analyzed using an unpaired Student’s t-test, while nonparametric data were analyzed using the Mann-Whitney U test or Kruskal-Wallis test. *P* values for group comparisons were FDR adjusted, according to Benjamin and Hochberg (1995). The corrected *P* values below 0.05 were considered statistically different. Data were expressed as means and standard error of the mean (SEM).

## RESULTS

### Growth Performance and Organ Measurements

The covariate effects of initial body weight (IBW), weaning age, and sex were not significant on performance parameters and therefore removed from the model. During the trial there were no mortalities. There was one pig (L) that was removed on day 41 of age due to health conditions and recommendation by the veterinarian. Body weight gain (ADG), feed intake (ADFI), and feed efficiency, FE (gain:feed) were statistically not different (*P* > 0.05) between pigs from parents with divergent EBV_FCR (Table 2). In addition, there were no differences in the relative organ weights nor the length of the small and large intestines between the two pig groups (*P* > 0.05, Table 3). Furthermore, the covariate effect of body weight of pigs whose organs were sampled was not significant (*P* > 0.05). There was, however, a trend for a larger heart (*P* = 0.065, Table 3) and larger lungs (*P* = 0.083, Table 3) as percentage of the respective pig’s weight, for the pig group from parents with the lower EBV_FCR (H group).

**Table 2.** Growth performance summary of nursery pigs fed the corn/soybean meal based diet. Least Square Means (LSMeans) for the H pigs and L pigs based on parental EBV_FCR were calculated and unpaired T-tests were used to test for significant differences between pig groups

BW, kg	H	L	SEM	*P* value
IBW, 21 d	7.0	6.7	0.15	0.0545
BW, 28 d	8.4^a^	8.2^b^	0.09	0.0067
BW, 42 d	16.5	16.1	0.34	0.3678
BW, 56 d	27.3	27.8	0.50	0.4662
ADG, g/d				
Day 28–42	575.1	567.0	13.63	0.6791
Day 42–56	778.0	812.3	12.27	0.0517
Day 28–56	1352.2	1379.5	17.75	0.2863
ADFI, g/d				
Day 28–42	888.5	903.1	22.50	0.6493
Day 42–56	1323.1	1354.8	48.66	0.6492
Day 28–56	2211.6	2257.9	63.83	0.6123
FE, gain:feed				
Day 28–42	0.64	0.62	0.011	0.2016
Day 42–56	0.59	0.60	0.029	0.4937
Day 28–56	0.61	0.61	0.017	0.8532

BW = body weight; IBW = initial body weight; ADG = average daily gain; ADFI = average daily feed intake; (N = 16 for each group). H = nursery pigs from parents with low EBV_FCR; L = nursery pigs from parents with high EBV_FCR; SEM = Standard error mean.

**Table 3.** Relative organ weights and organ length of nursery pigs from two groups from parents with different EBV_FCR (N = 8 for each group) measured at the end of feeding phase 2

Relative organ Weight, %	HE	LE	SEM	*P* value
Spleen	0.23	0.23	0.018	1.0000
Heart	0.60	0.52	0.030	0.0649
Lungs	1.21	1.05	0.061	0.0834
Kidneys	0.54	0.55	0.018	0.9226
Liver	3.22	3.47	0.100	0.1244
Stomach	0.75	0.74	0.047	0.8967
Small Intestine	4.11	3.70	0.280	0.3198
Large Intestine	2.13	2.02	0.210	0.7294
Organ length, m				
Small Intestine	18.0	17.4	0.60	0.5050
Large Intestine	4.0	3.2	0.35	0.1422

H = nursery pigs from parents with low EBV_FCR; L = nursery pigs from parents with high EBV_FCR; SEM = standard error mean (N = 8);.

### Nutrient and Energy Digestibility

Apparent total tract digestibility (ATTD) of nutrients and of gross energy were not different between the two pig groups (*P* > 0.05, Table 4). ATTD of crude protein, no fiber carbohydrates and of gross energy (GE) were higher than 88% regardless of pig group. ATTD of crude fiber and neutral detergent fiber (NDF) and acid digestible fiber (ADF) were higher than 40%, 68% and 69%, respectively, and not different between pig groups (*P* > 0.05).

**Table 4.** Apparent total tract digestibility (ATTD) of experimental diets fed to the nursery pigs

Item	HE	LE	SEM	*P* value
ATTD, %				
Dry matter	89.92	89.40	0.864	0.5554
Crude protein	89.40	88.70	1.174	0.5620
Crude fat	62.21	62.66	3.853	0.9093
Crude fiber	47.63	43.50	5.182	0.4410
Non-fiber carbohydrates	95.06	94.68	0.451	0.4094
NDF	69.36	68.38	2.136	0.6539
ADF	70.05	69.70	2.562	0.8927
Ash	75.86	75.61	1.674	0.8821
GE	90.06	89.56	0.904	0.5930

ATTD = apparent total tract digestibility; H = nursery pigs from parents with low EBV_FCR; L = nursery pigs from parents with high EBV_FCR; SEM = standard error mean; NDF = neutral detergent fiber; ADF = acid detergent fiber; GE = gross energy; N = 8.

### Taxonomic Classification and Diversity of Fecal Microbiota Related to Host Feed Efficiency

**Figure 1.**
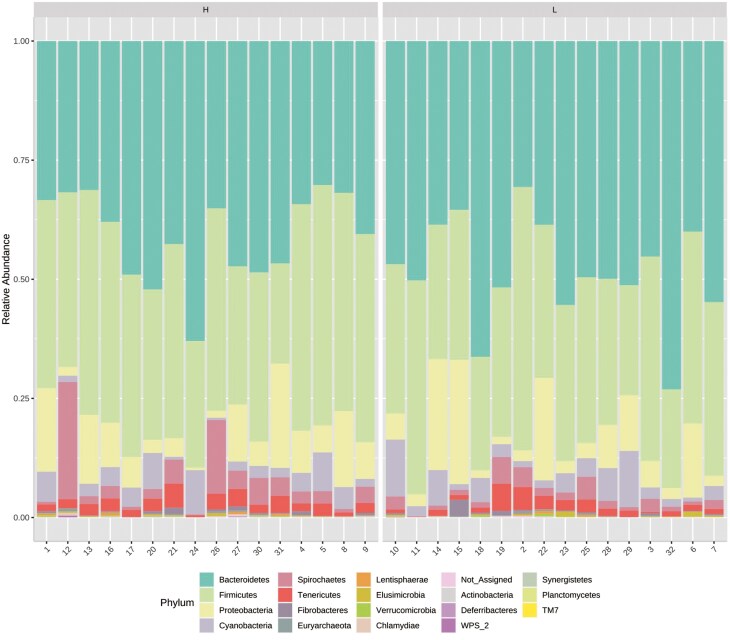
Relative abundance of bacteria of the most abundant taxa in the feces of nursery pigs at the phylum level. Numbers on the x-axis are pig IDs. N=16.

**Figure 2.**
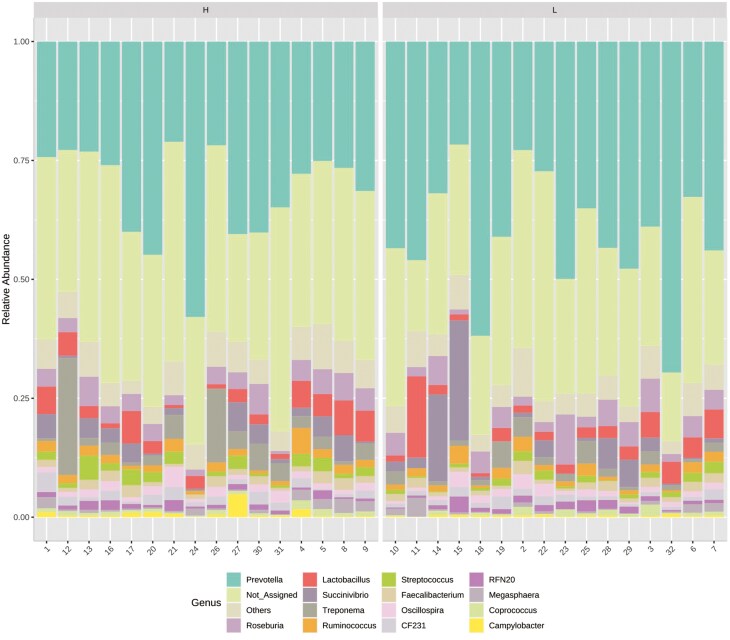
Relative abundance of bacteria of the most abundant taxa in the feces of nursery pigs at the genus level. Numbers on the x-axis are pig IDs. N=16.

At 56 d of age, hand grabbed fecal samples from each pen were collected for 16S rRNA sequencing. The number of sequences ranged from 70,575 to 193,788 (130,988 ± 22,996 sequences on average). After quality control with DADA2, we obtained 120,528 (± 21,059) reads per sample. With a 99% identity cutoff, the total number of Amplicon Sequence variants (ASVs) was 1960. Bacteroidetes, currently known as Bacteroidota and Firmicutes, currently known as Bacillota (Figure 1) were the two most abundant phyla regardless of the pig EBV_FCR group. *Prevotella* (Figure 2) was the most prevalent genus in both pig groups (36%); Statistical significances for abundant phyla and genera are reported in Table S1 and S2, respectively. At the phylum level, Bacteroidetes (45%) and Firmicutes (36%) were by far the most prevalent phyla, followed by Proteobacteria (9%), currently known as Pseudomonadota. At the genus level, *Prevotella* (36%), and *Roseburia* (4%) were the most abundant in both pig groups. *Lactobacillus*, *Ruminococcus*, and *Streptococcus* accounted for less than 4% of the total reads.

**Figure 3.**
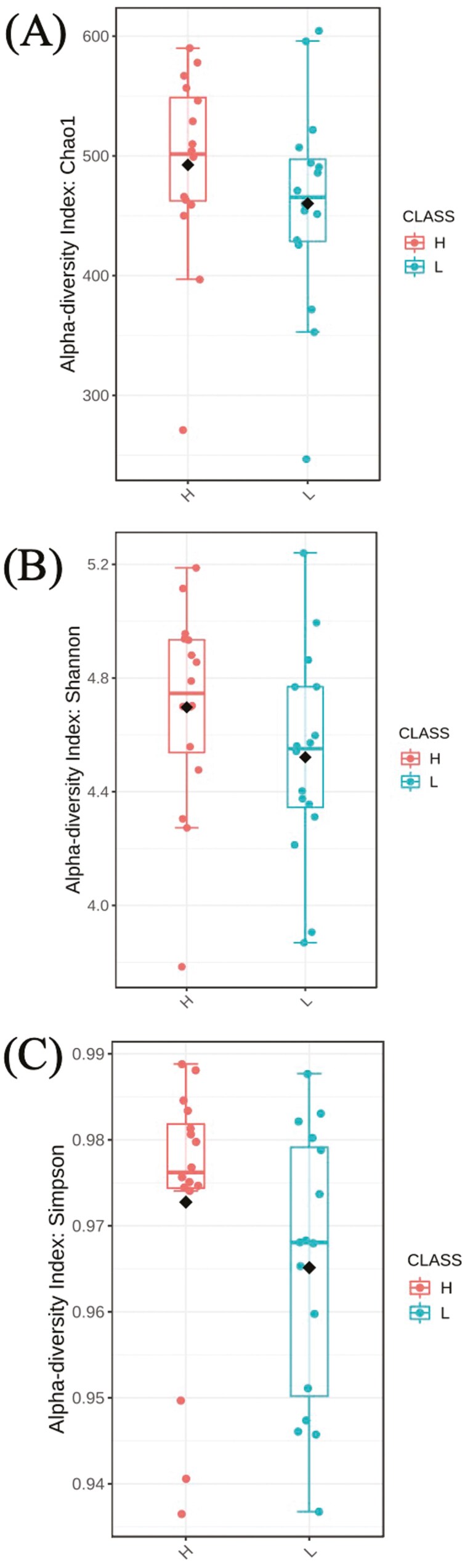
Alpha diversity in relation to host feed efficiency when bacteria aggregated at the feature level regarding to the species richness (A, Chao1 index, *P* = 0.183); species dominance (B, Shannon index, *P* = 0.110); and species richness and evenness (C, Simpson index, *P* = 0.160). H- Piglets from parents with low EBV_FCR; L–Piglets from parents with high EBV_FCR. N = 16.

**Figure 4.**
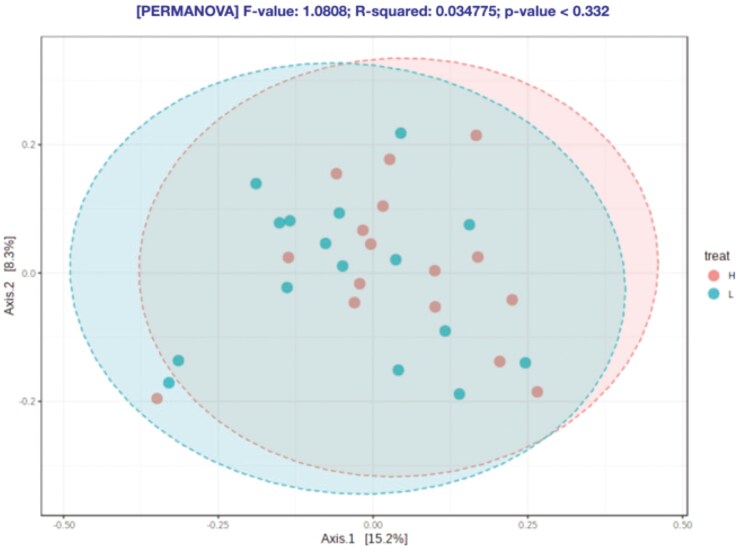
Beta diversity in relation to host feed efficiency at the feature level according to Bray-Curtis dissimilarity (PERMANOVA *P* < 0.332). Axes represent the two dimensions explaining the greatest proportion of variances in the communities for each analysis. H- Piglets from parents with low EBV_FCR; L – Piglets from parents with high EBV_FCR. N=16.

**Figure 5.**
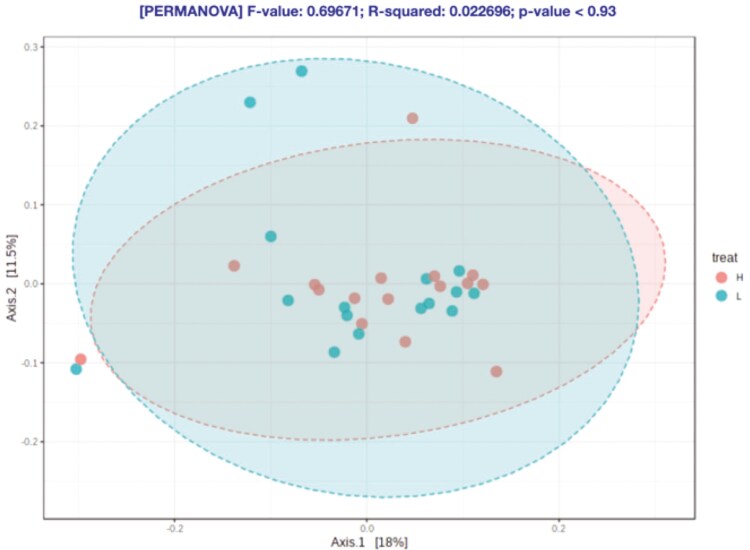
Beta diversity in relation to host feed efficiency at the feature level according to Unweighted Unifrac distances (PERMANOVA *P* < 0.930). Axes represent the two dimensions explaining the greatest proportion of variances in the communities for each analysis. H- Piglets from parents with low EBV_FCR; L – Piglets from parents with high EBV_FCR. N=16.

**Figure 6.**
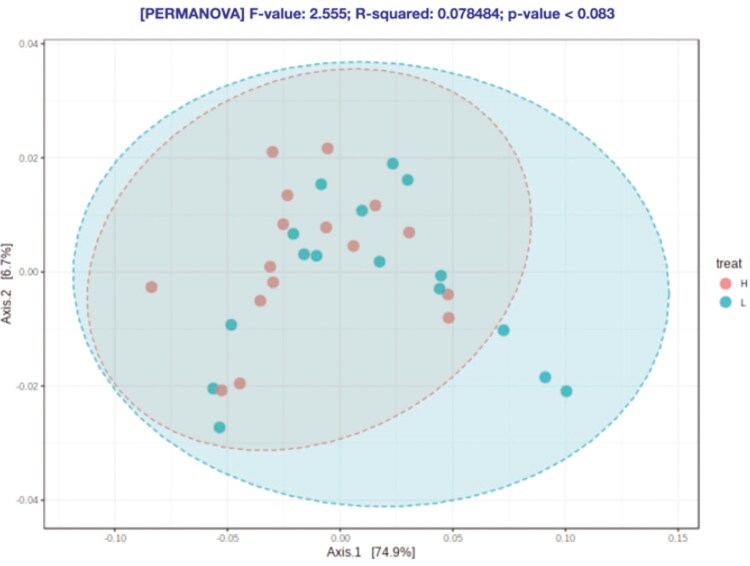
Beta diversity in relation to host feed efficiency at the feature level according to Weighted Unifrac distances (PERMANOVA *P* < 0.083). Axes represent the two dimensions explaining the greatest proportion of variances in the communities for each analysis. H- Piglets from parents with low EBV_FCR; L – Piglets from parents with high EBV_FCR. N=16.

At feature level, no statistical differences were observed for the alpha-diversity indexes (Figures 3–6) and beta-diversity (Figures 4–6). However, there was a trend for a higher beta diversity on fecal samples from pigs from parents with low EBV_FCR (Weighted Unifrac, PERMANOVA *P* < 0.083, Figure 6). Differential abundance analysis was done using the statistical model DESeq2 and the RLE data normalization method. At the genus level and FDR *P* value of 0.05, there were no significant differences between the two pig groups (Table S3).

### Predicted Functionality of Fecal Microbiota Related to Host Feed Efficiency

**Figure 7.**
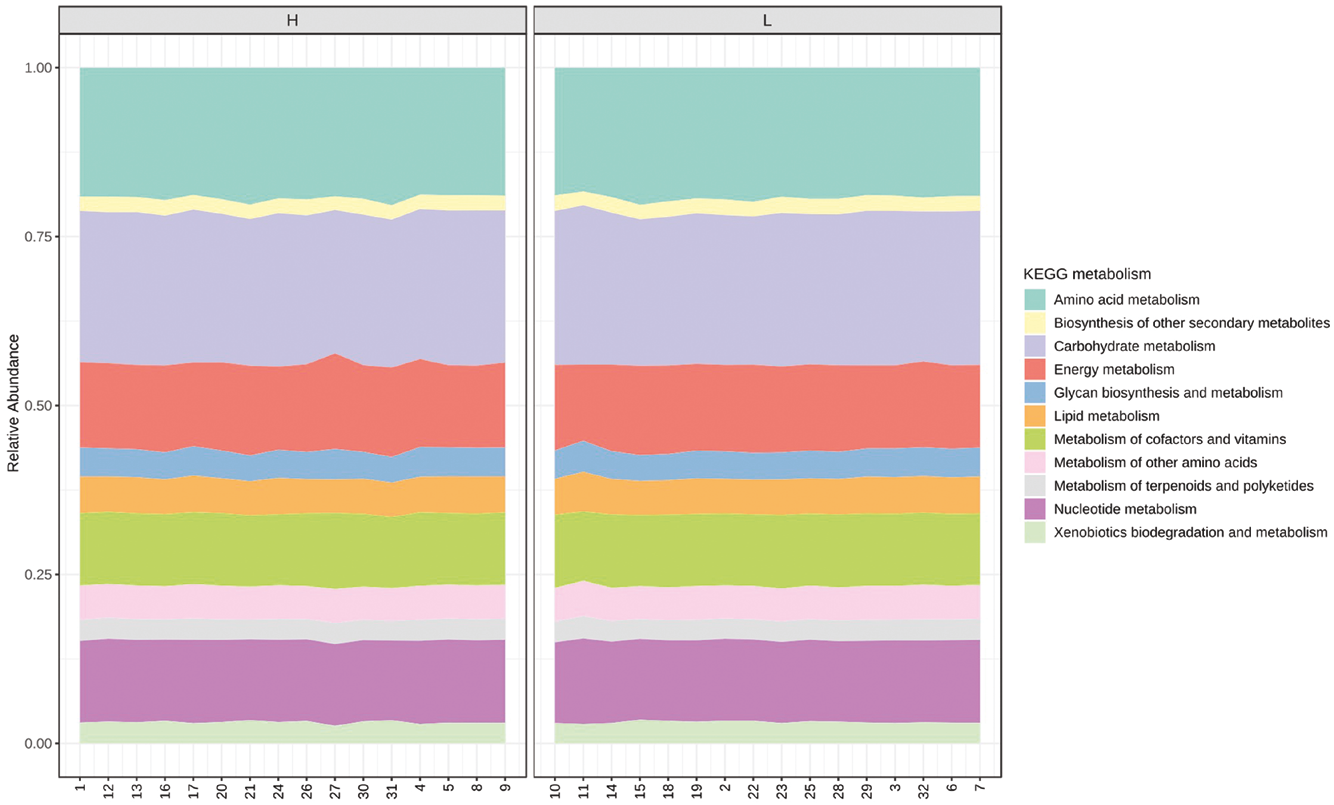
Predicted functionality of fecal microbiota related to host feed efficiency in terms of KEGG metabolism. The relative abundances of the various metabolism categories across all the samples are represented on the Y-axis. H- Piglets from parents with low EBV_FCR; L – Piglets from parents with high EBV_FCR. N = 16.

**Figure 8.**
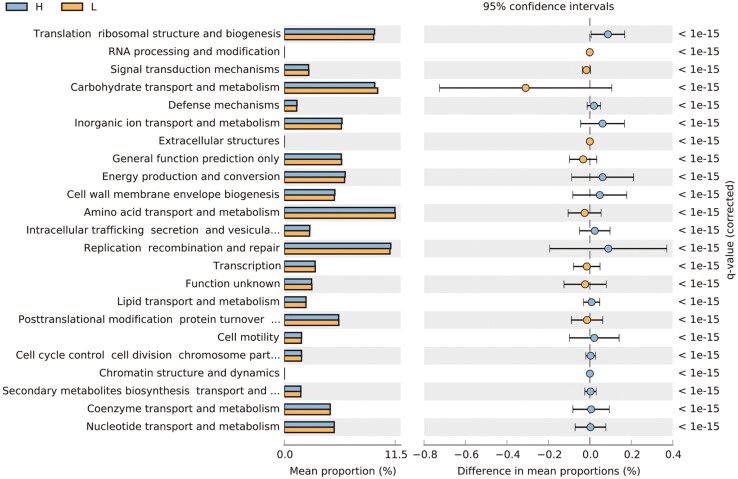
Predicted functionality of fecal microbiota related to host feed efficiency in terms of COG functional pathways. Blue and orange represent the post weaning group from parent with low EBV_FCR and high EBV_FCR, respectively. The samples are represented on the X-axis and separated based on metadata (pig FE group). The relative abundances of the various metabolism categories across all the samples are represented on the Y-axis. H- Piglets from parents with low EBV_FCR; L – Piglets from parents with high EBV_FCR. N = 16.

The R package, Tax4FUN was used to evaluate the functional profiles of the pig fecal microbiota for the two pig groups from parents with divergent EBV_FCR. The results indicated that 11 pathways, including carbohydrate, amino acids, and energy metabolism were not different between the two pig groups (Figures 7 and 8, Table S4) and it was dominated by carbohydrate and amino acid metabolism.

## DISCUSSION

### Growth Performance and Digestibility

Feed efficiency (FE) in pigs is a complex trait influenced by genetics, nutrition, management, and health status (Patience et al., 2015). This study examined the impact of pig genetics, specifically the Estimated Breeding Values for Feed Conversion Ratio (EBV_FCR) of their parents, on FE, growth, digestibility, and fecal microbiota. To isolate genetic effects, the study controlled for variables such as breed, sex, age, diet, and management practices. Results showed no significant differences in feeding behavior, growth, or mortality rates (0%) between pigs from high and low EBV_FCR parents. Also, both groups exhibited no signs of post-weaning diarrhea, indicating comparable physiological and health statuses. Nutrient and energy digestibility did not differ significantly between the two groups. Although pigs from parents with low EBV_FCR showed numerically higher apparent total tract digestibility (ATTD) of gross energy (GE), these differences were not statistically significant. This contrasts with findings by Vigors et al. (2016), who reported differences in nutrient digestibility associated with FE, and Harris et al. (2012), who observed higher digestibility in high FE pigs. Our results suggest that the high-quality diet used in this study, formulated to meet elevated nutrient standards, may have masked any potential differences in growth performance and nutrient digestibility. Differences between our study and previous research could also be attributed to the growth stage of the pigs, as we focused on nursery pigs, while the prior studies focused on grower pigs. These discrepancies suggest that genetic effects on FE and growth may become more evident in later growth stages, such as in grower-finisher pigs. This was observed in a companion study by Jin et al. (2022), where the same pigs as in our study were followed through the grower and finisher stages. In their studies, H pigs had significantly higher FE than L pigs at the grower and finisher stages, confirming the divergent FE.

At the nursery stage, it was encouraging to observe no growth disadvantages in the pigs from high EBV_FCR parents compared to those from low EBV_FCR parents, particularly during this critical period when pigs are highly vulnerable to infections, which could otherwise compromise growth and feed intake.

Regarding visceral organs, no significant differences were found in their relative sizes between the two groups. However, a trend toward larger hearts and lungs was observed in pigs from parents with lower EBV_FCR. This suggests that these pigs may possess anatomical adaptations that support higher metabolic rates and growth potential (Nyachoti et al., 2000).

### Microbiome in Relation to Feed Efficiency

Gut microbiota plays a crucial role in swine physiology, influencing energy harvest, nutrient digestion, and overall intestinal health, which in turn affects feed efficiency (FE) in pigs (McCormack et al., 2017). However, it remains unclear whether fecal microbiota in nursery pigs from parents with divergent estimated breeding values for feed conversion ratio (EBV_FCR) differ, or if specific gut microbiome adaptations in low (L) FE pigs could explain their similar growth and digestibility compared to high FE, i.e., H piglets. Research has identified certain bacterial taxa and microbiome functions linked to growth and FE. In the present study, we explored these aspects in H and L piglets. Gardiner et al. (2020) and Maltecca et al. (2020) highlighted bacteria involved in nutrient processing and those with anti-inflammatory properties as being associated with enhanced productivity. Taxa such as *Treponema*, *Roseburia*, and *Lactobacillus* have been linked to leaner phenotypes and improved FE (Gardiner et al., 2020). In contrast, our study found no differences in these genera or overall microbiota composition between pig groups, diverging from previous reports. This discrepancy could be attributed to factors such as diet, management practices, breed, sex, age, and gut site location (Tan et al., 2018; Bergamaschi et al., 2020; Gardiner et al., 2020; Fan and Kim, 2024). While previous studies focused on pigs selected based on extreme residual feed intake (RFI) values (Gardiner et al., 2020; Montagne et al., 2022), our study examined pigs from parents with divergent EBV_FCR, a trait with moderate to small heritability, which may partly explain the absence of observed differences. Nevertheless, a companion study to ours, followed these pigs through the grower-finisher stages by Jin et al. (2022), confirming the divergent FE.

Our study specifically targeted post-weaning nursery pigs, aiming to detect differences early, while other research has predominantly focused on grower-finisher pigs. Age is known to influence gut microbiota (Le Sciellour et al., 2019), which may explain the differences between our study and others. Weaning pigs are particularly vulnerable to gut dysbiosis, which could further contribute to varying results (Wei et al., 2021). However, our study identified Firmicutes and Bacteroidetes as the predominant phyla in fecal microbiota, consistent with previous findings (Yang et al., 2017; Wang et al., 2021). Core genera included *Prevotella*, *Ruminococcaceae*, and *Lactobacillus*, with *Prevotella* being particularly prevalent in the fecal samples from 56-d-old pigs. The absence of post-weaning diarrhea suggests these pigs did not experience microbial dysbiosis, which could be attributed in part to the premium diets used in our study. Verschuren et al. (2018) observed that diet can influence the relationship between FE and fecal microbiota composition, while sex might also modulate these dynamics in grower-finisher pigs, though its effect in nursery pigs is less well studied (Siebrits et al., 1986; Elbert et al., 2020). In our study, sex assignment to pens was random, and the covariate effect of sex on growth and fecal microbiota responses was not significant, suggesting that sex did not modulate these traits in nursery pigs.

Regarding microbiota diversity, we did not observe differences in alpha diversity at the feature level between pig groups; however, a trend towards higher alpha diversity at the genus level was noted in pigs from parents with lower EBV_FCR. This suggests that the relationship between microbiota diversity and FCR may vary depending on the taxonomic level examined. While no significant differences in beta diversity were found, there was a trend towards greater beta diversity in pigs from lower EBV_FCR parents, especially when considering phylogenetic distances and amplicon sequence variants (ASVs) using weighted UniFrac distances. Gardiner et al. (2020) also observed a trend towards greater bacterial diversity in more feed-efficient pigs, though inconsistencies across studies may stem from variations in age, sample types, and sample sizes. Studies by Wang et al. (2019) and Si et al. (2020) have reported age-dependent changes in alpha diversity, which could influence growth performance and FE. Le Sciellour et al. (2019) further highlighted age-related shifts in microbiota composition. Given these age-related changes, the impact of genetic selection for FE based on EBV_FCR on the gut microbiota of pigs at later grower-finisher stages warrants further investigation.

Differential abundance analysis using the DESeq2 method did not reveal any differentially abundant genera between the two pig groups, which was consistent with the genus-level compositional analysis.

Research has demonstrated a positive association between feed efficiency (FE) and specific Kyoto Encyclopedia of Genes and Genomes (KEGG) orthologies related to nitrogen and amino acid metabolism (Yang et al., 2017; Quan et al., 2019; Jiang et al., 2021). In our study, while KEGG orthologies associated with carbohydrate and amino acid metabolism were prevalent, no significant differences were observed between the pig groups, consistent with the findings of Lamendella et al. (2011). This underscores the variability in results across different studies. To gain a deeper understanding of how microbiota composition influences FE, further research is necessary, particularly in examining variations across different gut sections and ages. Two companion studies (Azevedo et al., 2025; Wu et al., 2025) are currently addressing these aspects, offering a more comprehensive view of the gut microbiota’s role in FE and body composition traits for the pigs used in our study.

## CONCLUSION

Despite selecting pigs from parents with low estimated breeding values (EBV) for feed conversion ratio (FCR), no significant improvement in growth performance or feed efficiency were observed at the nursery stage compared to pigs from parents with high EBV_FCR. Both groups exhibited similar nutrient and energy digestibility, suggesting that genetic background did not influence nutrient utilization. Furthermore, no differences in fecal microbiota composition or function were found between the groups, reinforcing the lack of observed differences in performance and feed efficiency. The study employed a high-quality diet with highly digestible ingredients, which may have obscured any potential genetic effects on growth and feed efficiency. Future research should examine the impact of genetic selection for FE using lower-quality diets or those incorporating more byproducts to more accurately assess the practical benefits of genetic selection in swine production systems.

## Supplementary Material

txaf026_suppl_Supplementary_Tables

## Data Availability

The datasets supporting the conclusions of this article will be deposited in the NCBI Sequence Read Archive database under the accession number PRJNA1160217 (microbiota raw sequencing data). All other data are contained within the main manuscript.
