# Supplementary material for: Feed efficiency and fecal microbiome of nursery pigs from parents with divergent breeding value for feed conversion ratio
Source: Transl Anim Sci. 2025 Feb 20;9:txaf026. doi: 10.1093/tas/txaf026 (PMC12012672; doi:10.1093/tas/txaf026)
Supplement: txaf026_suppl_Supplementary_Tables [file txaf026_suppl_supplementary_tables.docx]

**Table S1.** Statistics, *P* values and FDR adjusted *P* values for genotype effects on microbiota at the phylum level

|  | *P* values | FDR | Statistics |
| --- | --- | --- | --- |
| *Chlamydiae* | 0.009218 | 0.1567 | 197.5 |
| *Spirochaetes* | 0.10164 | 0.4522 | 172 |
| *Firmicutes* | 0.10997 | 0.4522 | 171 |
| *Lentisphaerae* | 0.12684 | 0.4522 | 169 |
| *Tenericutes* | 0.13808 | 0.4522 | 168 |
| *Bacteroidetes* | 0.15961 | 0.4522 | 90 |
| *Deferribacteres* | 0.19449 | 0.4723 | 162 |
| *Actinobacteria* | 0.41755 | 0.7887 | 106 |
| *Verrucomicrobia* | 0.47243 | 0.8031 | 147.5 |
| *Proteobacteria* | 0.5641 | 0.8718 | 144 |
| *Synergistetes* | 0.6166 | 0.8735 | 141.5 |
| *Fibrobacteres* | 0.79124 | 0.9605 | 135.5 |
| *Elusimicrobia* | 0.82213 | 0.9605 | 122 |
| *Euryarchaeota* | 0.88016 | 0.9605 | 123.5 |
| *Cyanobacteria* | 0.926 | 0.9605 | 125 |
| *TM7* | 0.96047 | 0.9605 | 129.5 |

FDR = False Discovery rate adjusted P values. N = 16.

**Table S2.** Statistics, *P* values and FDR adjusted *P* values for genotype effects on microbiota at the genus level

|  | *P* values | FDR | Statistics |
| --- | --- | --- | --- |
| *Chlamydia* | 0.009218 | 0.5807 | 197.5 |
| *Prevotella* | 0.056216 | 0.9564 | 77 |
| *Streptococcus* | 0.079568 | 0.9564 | 175 |
| *Lachnospira* | 0.079654 | 0.9564 | 175 |
| *Treponema* | 0.10164 | 0.9564 | 172 |
| *SMB53* | 0.1053 | 0.9564 | 171 |
| *L7A* | 0.12548 | 0.9564 | 169 |
| *Mogibacterium* | 0.1929 | 0.9564 | 163 |
| *Mucispirillum* | 0.19449 | 0.9564 | 162 |
| *CF231* | 0.22401 | 0.9564 | 161 |
| *Peptococcus* | 0.23438 | 0.9564 | 160 |
| *Acidaminococcus* | 0.2401 | 0.9564 | 159.5 |
| *Catenibacterium* | 0.2503 | 0.9564 | 97 |
| *Campylobacter* | 0.25423 | 0.9564 | 159 |
| *Turicibacter* | 0.27651 | 0.9564 | 155.5 |
| *Butyricicoccus* | 0.29153 | 0.9564 | 104.5 |
| *Megasphaera* | 0.30452 | 0.9564 | 156 |
| *p_75_a5* | 0.30869 | 0.9564 | 155.5 |
| *Lachnobacterium* | 0.31033 | 0.9564 | 154.5 |
| *Collinsella* | 0.34067 | 0.9564 | 102.5 |
| *Dialister* | 0.34472 | 0.9564 | 152.5 |
| *Helicobacter* | 0.36178 | 0.9564 | 107 |
| *Dorea* | 0.36567 | 0.9564 | 103.5 |
| *Victivallis* | 0.38627 | 0.9564 | 150.5 |
| *Oscillospira* | 0.42302 | 0.9564 | 150 |
| *Coprococcus* | 0.44504 | 0.9564 | 149 |
| *Methanosphaera* | 0.45269 | 0.9564 | 148 |
| *Gemmiger* | 0.46767 | 0.9564 | 148 |
| *02d06* | 0.4739 | 0.9564 | 147.5 |
| *Mitsuokella* | 0.49748 | 0.9564 | 146.5 |
| *Methanobrevibacter* | 0.51009 | 0.9564 | 145.5 |
| *Faecalibacterium* | 0.51475 | 0.9564 | 146 |
| *Sutterella* | 0.53395 | 0.9564 | 111 |
| *Oxalobacter* | 0.55751 | 0.9564 | 144 |
| *Lactobacillus* | 0.5641 | 0.9564 | 144 |
| *Eubacterium* | 0.59765 | 0.9564 | 113.5 |
| *Asteroleplasma* | 0.6441 | 0.9564 | 115.5 |
| *Clostridium* | 0.65105 | 0.9564 | 115.5 |
| *Slackia* | 0.69354 | 0.9564 | 138.5 |
| *vadinCA11* | 0.70397 | 0.9564 | 117.5 |
| *Sphaerochaeta* | 0.72747 | 0.9564 | 118.5 |
| *Anaerovibrio* | 0.75205 | 0.9564 | 137 |
| *Butyricimonas* | 0.75458 | 0.9564 | 134.5 |
| *Succinivibrio* | 0.78044 | 0.9564 | 136 |
| *YRC22* | 0.78061 | 0.9564 | 120.5 |
| *Fibrobacter* | 0.79124 | 0.9564 | 135.5 |
| *Subdoligranulum* | 0.79379 | 0.9564 | 122 |
| *Blautia* | 0.80913 | 0.9564 | 135 |
| *RFN20* | 0.80913 | 0.9564 | 121 |
| *Roseburia* | 0.80913 | 0.9564 | 121 |
| *Pseudobutyrivibrio* | 0.83491 | 0.9564 | 132.5 |
| *Butyrivibrio* | 0.85047 | 0.9564 | 133.5 |
| *Anaerostipes* | 0.86063 | 0.9564 | 123 |
| *Defluviitalea* | 0.86249 | 0.9564 | 133 |
| *Desulfovibrio* | 0.86462 | 0.9564 | 133 |
| *Parabacteroides* | 0.86529 | 0.9564 | 123 |
| *Bacteroides* | 0.91372 | 0.9876 | 125 |
| *Bulleidia* | 0.92493 | 0.9876 | 131 |
| *Ruminococcus* | 0.95556 | 1 | 130 |
| *rc4_4* | 0.9827 | 1 | 129 |
| *Phascolarctobacterium* | 0.98518 | 1 | 129 |
| *Oribacterium* | 1 | 1 | 128 |

FDR = False Discovery rate adjusted P values. N = 16.

**Table S3.** Statistics, *P* values and FDR adjusted *P* values for genotype effects from DESeq2 analysis at the genus level. Only P values lower than 0.1 are presented.

| Genus | log2FC | lfcSE | Pvalues | FDR |
| --- | --- | --- | --- | --- |
| *Chlamydia* | 1.8051 | 0.58406 | 0.001998 | 0.15585 |
| *Dorea* | -0.68975 | 0.31549 | 0.028795 | 0.63536 |
| *Campylobacter* | 0.91185 | 0.45375 | 0.044476 | 0.63536 |
| *Turicibacter* | 2.2515 | 1.1299 | 0.046306 | 0.63536 |
| *Treponema* | 0.92463 | 0.46927 | 0.048798 | 0.63536 |
| *SMB53* | 1.799 | 0.91332 | 0.048874 | 0.63536 |
| *Mucispirillum* | 1.6745 | 0.93279 | 0.072635 | 0.80936 |

Log2FC =log2 fold change; lfcSE = log2 fold change standard error; FDR= false discovery rate P value; N = 16.

**Table S4.** Statistics, *P* values and FDR adjusted *P* values for genotype effects on functionality at the genus level

|  | *P* values | FDR | Statistics |
| --- | --- | --- | --- |
| Translation ribosomal structure and biogenesis | 0.03874 | 0.4492 | 183 |
| RNA processing and modification | 0.04084 | 0.4492 | 73.5 |
| Inorganic ion transport and metabolism | 0.19637 | 0.5915 | 163 |
| Nucleotide transport and metabolism | 0.20987 | 0.5915 | 162 |
| Carbohydrate transport and metabolism | 0.25423 | 0.5915 | 97 |
| Defense mechanisms | 0.27033 | 0.5915 | 158 |
| Chromatin structure and dynamics | 0.27365 | 0.5915 | 157.5 |
| Cell cycle control cell division chromosome partitioning | 0.28267 | 0.5915 | 157 |
| Secondary metabolites biosynthesis transport and catabolism | 0.28709 | 0.5915 | 157 |
| Signal transduction mechanisms | 0.28709 | 0.5915 | 99 |
| Cell wall membrane envelope biogenesis | 0.30452 | 0.5915 | 156 |
| Replication recombination and repair | 0.32262 | 0.5915 | 155 |
| General function prediction only | 0.53915 | 0.9124 | 111 |
| Energy production and conversion | 0.64202 | 0.9392 | 141 |
| Amino acid transport and metabolism | 0.72398 | 0.9392 | 118 |
| Posttranslational modification protein turnover chaperones | 0.72398 | 0.9392 | 118 |
| Coenzyme transport and metabolism | 0.78044 | 0.9392 | 120 |
| Intracellular trafficking secretion and vesicular transport | 0.80913 | 0.9392 | 135 |
| Cell motility | 0.83577 | 0.9392 | 122 |
| Lipid transport and metabolism | 0.89653 | 0.9392 | 132 |
| Transcription | 0.89653 | 0.9392 | 132 |
| Function unknown | 0.98518 | 0.9852 | 129 |

FDR = False Discovery rate adjusted P values. N = 16.
